# Three-Year Follow-Up of children and adolescents with OCD Who Did Not Respond to Initial Cognitive-Behavioral Therapy (CBT): Outcomes of Continued CBT vs. Sertraline

**DOI:** 10.1007/s00787-026-03009-3

**Published:** 2026-03-19

**Authors:** Gudmundur Skarphedinsson, Bernhard Weidle, Nor Christian Torp, Davíð R. M. A. Højgaard, Sanne Jensen, Karin Melin, Katja Anna Hybel, Per Hove Thomsen, Judith B. Nissen, Tord Ivarsson

**Affiliations:** 1 Faculty of Psychology, University of Iceland, Reykjavik, Iceland; 2 Regional Centre for Child and Youth Mental Health and Child Welfare, Norwegian University of Science and Technology, Trondheim, Norway; 3 St. Olav’s University Hospital, Trondheim, Norway; 4 Department of Child and Adolescent Psychiatry, Division of Mental Health and Addiction, Vestre Viken Hospital, Drammen, Norway; 5 Division of Mental Health and Addiction Services, Akershus University Hospital, Oslo, Norway; 6 Department of Clinical Medicine, Health, Aarhus University, Aarhus, Denmark; 7 Department of Child and Adolescent Psychiatry, Aarhus University Hospital, Psychiatry, Aarhus, Denmark; 8 Gillberg Neuropsychiatry Centre, Institute of Neuroscience and Physiology, Sahlgrenska Academy, University of Gothenburg, Gothenburg, Sweden; 9 Department of Pediatric Neurology and Psychiatry, Region Västra Götaland, Sahlgrenska University Hospital, Gothenburg, Sweden; 10 Institute of Neuroscience and Physiology, University of Gothenburg, Gothenburg, Sweden

**Keywords:** Obsessive-compulsive disorder, Children and adolescents, Cognitive-behavioral therapy, Sertraline, Selective serotonin reuptake inhibitors, Follow-up, Randomized controlled trial

## Abstract

Cognitive-behavioral therapy (CBT) is the first-line treatment for pediatric obsessive-compulsive disorder (OCD), with selective serotonin reuptake inhibitors (SSRIs) recommended for non-responders. However, evidence on the long-term outcomes of these interventions is limited. This study examines outcomes over three years for youth with OCD who did not respond to initial CBT and were randomized to either continued CBT or sertraline. Fifty children and adolescents aged 7–17 years, with OCD according to DSM-IV criteria, were included in this randomized controlled trial conducted across Denmark, Sweden, and Norway. Participants who did not respond to 14 weeks of CBT were randomized to 16 weeks of continued CBT or sertraline. Outcomes included the Children’s Yale-Brown Obsessive-Compulsive Scale (CY-BOCS) total scores and clinical remission (CY-BOCS < 11) at all follow-up points: 12, 24, and 36 months. Linear mixed effects showed significant reduction in the CY-BOCS total score over time across treatments. Planned pairwise comparison of the CY-BOCS total score did not reveal a significant difference between the treatments at 12-, 24-, or 36-month follow-up. At the three-year follow-up, 92% of participants showed considerable improvement: 77% achieved clinical remission and another 15% showed only mild OCD symptoms (CY-BOCS < 16). No serious AEs occurred. Continued CBT and sertraline both demonstrate long-term effectiveness for children and adolescents with OCD who do not respond to initial CBT, with durable outcomes observed across groups. These findings support the stepped-care model and the importance of individualized treatment planning, as both approaches can lead to sustained improvement.

**Supplementary Information:**

The online version contains supplementary material available at 10.1007/s00787-026-03009-3.

## Introduction

Obsessive-compulsive disorder (OCD) is characterized by the presence of obsessions and compulsions [1] with a population prevalence of 1–3% [2–6]. OCD often leads to significant distress and severe functional impairment throughout childhood and adulthood [7–10].

Clinical expert guidelines recommend cognitive behavioral therapy (CBT) and selective serotonin reuptake inhibitors (SSRIs) as effective treatments for OCD in children [11–14]. Both treatments have well-documented short-term effects, typically observed within 8 to 16 weeks. A systematic review found that remission rates for CBT and SSRIs are 53% and 24%, respectively [15]. According to European expert guidelines, CBT is recommended as the first-line treatment for children with OCD, while SSRIs should only be considered if the child does not respond to CBT. These guidelines are supported by multiple studies and publications [12, 14, 16].

Despite the availability of effective treatments, the long-term outcomes for patients with OCD are still a concern, especially for those who do not respond to initial treatment. Therefore, examining the long-term trajectories of patients receiving treatment is critically important. Initial longitudinal studies on the prognosis of youth with OCD indicated poor outcomes, with little improvement over time [17]. However, more recent studies have shown more promising results. Several studies conducted over the past decade have shown encouraging outcomes 3–7 years after treatment [18–21], with up to 62% of patients with OCD achieving remission 1–16 years after treatment. Additionally, these studies indicate that most patients continue to show improvement even after treatment has ended [22].

While the results of these studies are promising, it is important to note that recovery from OCD may be a complex and lengthy process. Treatment pathways differ between patients, and some patients may need multiple courses of therapy or a combination of CBT and medications to achieve and sustain remission. Additionally, individuals with OCD may continue to experience symptoms at varying degrees even after treatment and might require ongoing support and monitoring to prevent relapse. In conclusion, tracking long-time trajectories is essential to better understanding prognosis and the effectiveness of treatment.

The Nordic Long-Term OCD Treatment Study (NordLOTS) was designed to evaluate the effectiveness of first-line CBT for youth with OCD. Additionally, it aimed to assess the outcomes of non-responders to CBT after randomizing them to either 16 weeks of continued CBT or sertraline [23–25]. The immediate outcome showed no significant differences between the groups, but both treatments demonstrated large and significant within-group effect sizes. Furthermore, no serious adverse events (AEs) were reported in the group that received sertraline [26].

The previously published long-term results have highlighted the effectiveness of this stepped-care model. At the end of the three-year follow-up period, 90% of patients were classified as responders, and 73% reached clinical remission [20]. However, only 36.4% of all patients remained in full remission throughout the follow-up period [27], indicating that while a majority achieved clinical remission at some point, maintaining it continuously was less common.

The current study aims to extend our previous research and examine the long-term outcomes of children who were randomized to receive sertraline or continued CBT. We conducted a three-year follow-up with all patients after their initial CBT treatment, providing sertraline also to those who did not respond to a second round of CBT. All patients were evaluated at six, twelve, twenty, and thirty-six months after their initial CBT treatment, and the adverse events of patients who received sertraline were assessed.

### Aims

The study aims to examine the long-term outcomes of children and adolescents who did not respond to first-line CBT and were then randomized to sertraline or continued CBT. We also follow adverse events in the sertraline group. The specific aims are (1) to report on the long-term outcomes of the two treatment groups, and (2) to describe long-term medication use and reported adverse events (AEs).

## Method

### Study design and procedures

NordLOTS is a multi-center, randomized clinical trial that aims to evaluate the effectiveness of continued exposure-based CBT or pharmacotherapy with sertraline for the treatment of moderate to severe OCD in children and adolescents. The study design and methods have previously been described in detail elsewhere [23–26].

The study consisted of three main treatment steps. In Step 1, all participants received 14 weeks of exposure-based CBT. Non-responders to Step 1 (indicated by a score greater than 15 on the Children’s Yale-Brown Obsessive-Compulsive Scale (CY-BOCS)) were randomized to receive either 16 weeks of continued CBT or pharmacotherapy with sertraline in Step 2. Participants (*n* = 10) who were randomized to continued CBT but did not respond (CY-BOCS > 15) were also offered sertraline treatment. One participant declined medication, while nine accepted this treatment [28]. Treatment outcomes were evaluated 6 months, 1 year, 2 years, and 3 years after completing CBT in Step 1 [20, 24]. Randomization was stratified by gender and the presence of a tic disorder, centrally conducted at the coordinating center in Oslo, for all participating sites to prevent allocation prediction. Informed consent was obtained from the participants’ parents or guardians and from the participants if they were 11 years or older. The study was approved by the Norwegian, Swedish, and Danish Committees for Medical and Health Research Ethics and the Medical Products Agencies. It was registered with Current Controlled Trials (www.controlled-trials.com ISRCTN66385119). No industry funding was provided. Funding was applied for at each national site, resulting in the total study receiving financial support from national funders and some central funding. A list of all funding sources has been published elsewhere [24].

### Participants

The study was conducted in 19 clinics in Denmark, Norway, and Sweden, and involved 269 children and adolescents between the ages of 7 and 17 years at the start of the study [23]. Participants were recruited through referrals and were diagnosed with OCD and any comorbid disorders using the Schedule for Affective Disorders and Schizophrenia for School-Age Children Present and Lifetime Version (K-SADS-PL) [29]. To be eligible for step 2, participants had to be rated as non-responders (CY-BOCS > 15) at the end of step 1 and agreeing to randomization to either continued CBT or pharmacotherapy with sertraline. A total of 50 children and adolescents were included in Step 2 [26].

### Measures

The CY-BOCS, a widely used clinician-administered tool for evaluating obsessions and compulsions [30], was used to assess treatment response. The CY-BOCS assesses the severity of obsessions and compulsions separately, providing scores ranging from 0 to 20 for each, resulting in a composite score of 0 to 40. Its psychometric properties are considered adequate [30, 31] and interrater agreement was found to be ICC = 0.92 in the NordLOTS sample [23]. CY-BOCS assessments were conducted at baseline and after 7 and 14 weeks in Step 1, after 22 and 30 weeks in Step 2, and at 38 and 46 weeks for those receiving sertraline post-Step 2 CBT. Follow-up assessments were done 6 months, 1 year, 2 years, and 3 years after completing Step 1. Treatment response was defined as a total CY-BOCS score of less than 16 [23], while clinical remission was indicated by a total CY-BOCS score of less than 11 [23, 32, 33]. All outcome assessments were conducted by independent evaluators (IEs) who were not involved in treatment delivery. The IEs were not blinded to treatment allocation at any assessment point, including Step 2 and all follow-up evaluations. This reflects the pragmatic design of the study, in which treatments were delivered within routine clinical settings and blinding of IEs was not feasible.

The *Schedule for Affective Disorders and Schizophrenia – Present and Lifetime version (K-SADS-PL)* was used to confirm OCD and comorbid diagnoses. K-SADS is a standardized diagnostic interview for the assessment of psychiatric disorders in children and adolescents according to DSM-IV criteria [29]. The K-SADS-PL has excellent inter-rater reliability [25, 34, 36] and convergent and divergent validity [35, 37]. Symptoms can be classified as “not present,” “possible,” “in remission,” or “certain.” In this study, diagnoses were based on symptoms classified as certain only.

The Children’s Global Assessment Scale (CGAS) is a clinician-rated measure of overall functioning in children and adolescents, scored from 1 to 100, with lower scores indicating greater impairment. The scale has demonstrated acceptable test-rest and inter-rater reliability [38] and discriminant and concurrent validity [39].

### Treatment

Continued CBT in Step 2 consisted of 10 additional sessions over 16 weeks, designed to address barriers that may have affected the success of Step 1. Participants received CBT using the same principles as in Step 1. However, the therapist assessed and tailored the treatment based on individual factors, such as delayed engagement in exposure exercises, the predominant occurrence of symptoms in specific settings, low motivation or expectations for improvement, and problematic family dynamics. The sessions were 90 min long, with the first hour focused on individual E/RP and the last half hour on family sessions, conducted similarly to the approach by Piacentini and colleagues [33]. The format of Step 2 CBT was less manualized and more tailored to individual needs while remaining consistent with the principles of effective E/RP CBT [26].

Pharmacotherapy with sertraline involved six sessions over 16 weeks, using a manual based on the Pediatric OCD Treatment Study (POTS) [32]. The treatment started with a dose of 25 mg/day and was gradually titrated up to 100 mg/day by week 4, and gradually increasing to a maximum dose of 200 mg/day if the response was deemed inadequate. Symptom severity and adverse events were monitored during each session, and the dose was reduced if necessary. Sertraline was chosen for treatment because it was the only approved SSRI for OCD in children and adolescents in Denmark, Sweden, and Norway, and it is equally effective as other SSRIs. Clinical support was also provided during treatment to encourage the exposure tasks learned during step 1, but no new tasks were introduced. The pharmacotherapists followed a standardized script and asked the child and parent(s) about resistance to compulsions, activities the child would engage in once they improved, the active use of treatment techniques, exposure outcomes, and any concerns related to the medication [26]. The following criteria for reducing the maintenance dose were assessed at every visit: if the patient was very much improved (Clinical Global Impression Improvement = 6), if the OCD illness was subclinical or in clinical remission (Clinical Global Impression Severity = 0 or 1) [40], and if CY-BOCS scores were ≤ 10 points. If a reduced dose led to worsened OCD symptoms or functioning, the dosage was increased back to the full level. If a patient met termination criteria during medication follow-up, namely 6 months of subclinical OCD or remission, sertraline was reduced by 25% every one to two weeks until a dose of 25 mg was reached. This dose was maintained for one or two weeks to prevent withdrawal symptoms, after which the medication was discontinued.

AEs were monitored at each visit using the same structured checklist as in the initial paper of the immediate outcomes [26], adapted from Kutcher [41]. The checklist was completed jointly by the child and parent and reviewed by the treating physician to determine frequency, severity and impairment associated with each event. Serious adverse events were reported to the principal investigator (TI).

### Statistical analysis

For the linear mixed effects (LME) models analyses, our approach was guided by the intent-to-treat principle, which states that all patients should be included in the analysis regardless of their adherence to the treatment protocol. Thus, all 50 participants who entered step 2 treatment were included in the LME analyses. In the long-term outcome analyses, participants who crossed over to sertraline after non-response to continued CBT were analyzed according to their original Step 2 randomization, consistent with the intention-to-treat principle. Baseline demographic and clinical variables compared participants with missing and non-missing data, and none of these comparisons showed significant differences. Furthermore, no differences were found when estimating treatment outcomes. Based on these results, the LME analyses were conducted under the assumption that data were missing at random (MAR).

For the categorical outcome variables (mild OCD, CYBOCS < 16; and clinical remission, CY-BOCS < 11), analyses were conducted using completers-only data. At each assessment point, the proportion of participants meeting each criterion was calculated separately for the continued CBT and sertraline groups, and group differences were evaluated using chi-square tests (or Fisher’s exact test when cell sizes were small). No imputation procedures were applied to categorical outcomes, as the small sample size and event frequencies made multiple imputation unreliable for these variables. All tests were two-tailed, and *p* < 0.05 was considered to indicate statistical significance.

Adverse events (AE) and medication data (dose, duration, and AE frequences) were summarized descriptively based on available data only.

## Results

### Recruitment and retention

**Fig. 1:**
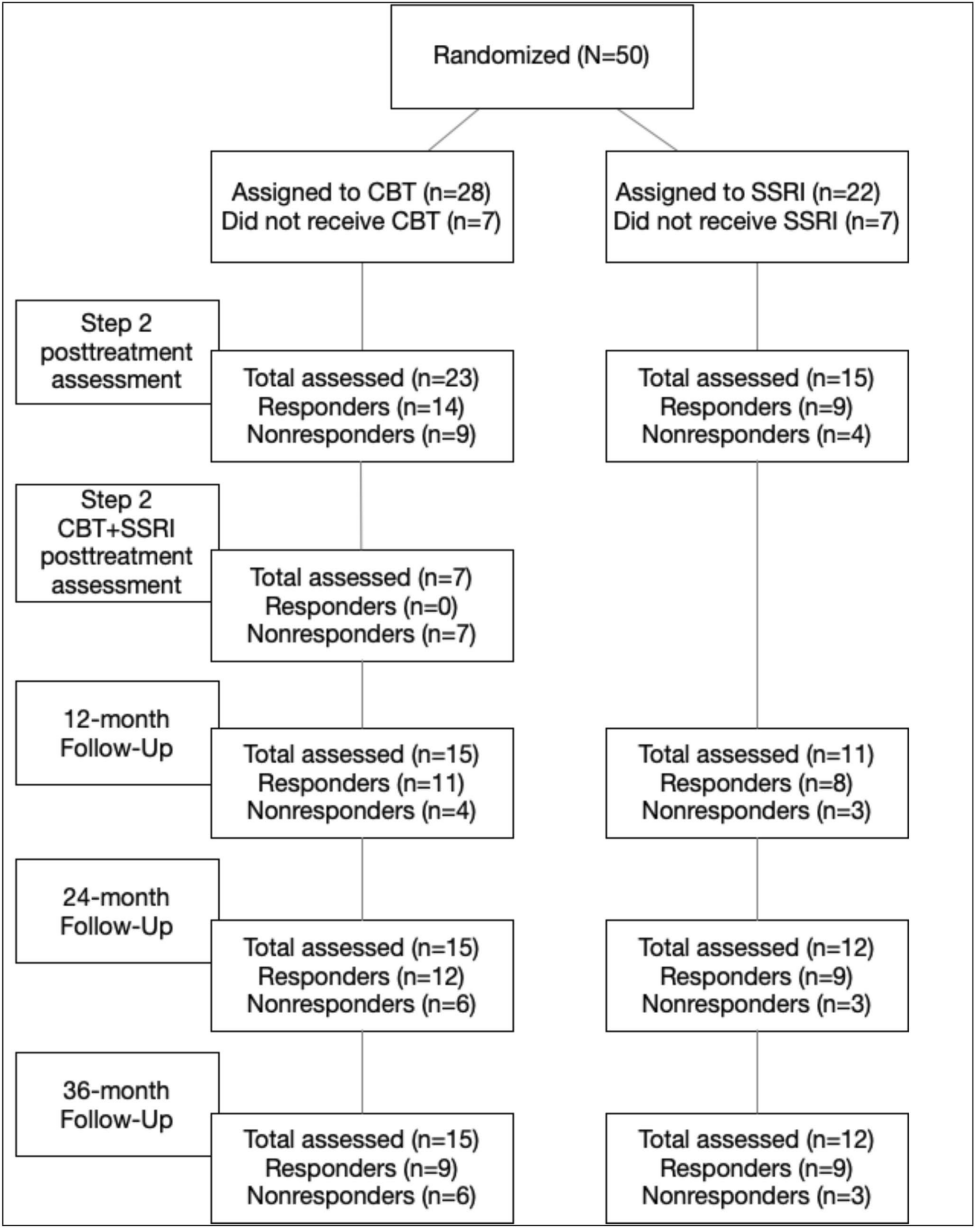
Step 2 Follow-Up Flowchart

We randomized 54 participants in step 2. Four participants who were assigned to sertraline were re-evaluated using the CY-BOCS due to a treatment delay of more than four weeks. These four participants scored below 16 on the CY-BOCS reassessment and were therefore not eligible for Step 2 treatment, but were considered Step 1 treatment responders. A total of 50 participants were included. Attrition for follow-up assessments was 24% (*n* = 12) at step 2 post-treatment, 48% (*n* = 24) at 1-year follow-up, 46% (*n* = 23) at 2-year follow-up, and 46% (*n* = 23) at 3-year follow-up (see Fig. 1).

### Patient characteristics

The characteristics of the Step 2 participants are detailed in Table 1. On average, there was a 16.4% decrease in the total CY-BOCS score from the initial assessment to week 14, with a standard deviation of 19.8%. Among the participants, 32.0% (*n* = 16) experienced either no significant change in their CY-BOCS scores or a reduction of less than 10% by the beginning of week 14. Additionally, 42.0% (*n* = 21) observed a modest decrease in their CY-BOCS scores of less than 30% during this period, while 26.0% (*n* = 13) achieved a reduction ranging from 30% to 47%. Analysis showed no significant statistical differences between the groups, with a *t*-score of − 1.01 and a *p*-value of 0.317. Compliance with the initial CBT regimen was deemed satisfactory, with 48% (*n* = 24) demonstrating “good” or “excellent” adherence and 76% (*n* = 38) also showing high levels of engagement.

**Table 1:** Baseline demographic and clinical characteristics by treatment group in NordLOTS Step 2

Characteristics	Sertraline (*n* = 22)	CBT (*n* = 28)	Total (*N* = 50)
Sex, No. (%)			
Male	11 (50.0)	13 (46.4)	24 (48.0)
Female	11 (50.0)	15 (53.6)	26 (52.0)
**Age**, mean (SD) in years	14.1 (2.8)	14.0 (2.8)	14.0 (2.7)
**Family status**			
Biological parents living together	15 (68.2)	16 (57.1)	31 (62.0)
Divorced	7 (31.8)	12 (42.9)	19 (38.0)
**SES**			
High	14 (63.6)	16 (57.1)	30 (62.5)
Low	8 (36.4)	10 (35.7)	18 (37.5)
**Ethnicity**			
At least one Scandinavian parent	22 (100)	27 (96.4)	49 (98.0)
**Scalar variables**,** mean (SD)**			
CYBOCS Total Score Week 0	25.2 (5.0)	27.3 (5.9)	26.4 (5.6)
CYBOCS Total Score Week 13	21.1 (3.7)	21.3 (4.0)	21.3 (3.8)
CGAS Week 0	52.8 (10.3)	52.3 (6.4)	52.5 (8.2)
CGAS Week 13	58.1 (10.5)	58.6 (8.2)	58.4 (9.2)
**Psychiatric comorbid disorders**,** No. (%)**			
Any depressive disorders	0 (0)	3 (10.7)	3 (6.0)
Any anxiety disorders	4 (18.2)	8 (28.6)	12 (24.0)
ADHD	2 (9.1)	5 (17.9)	7 (14.0)
ODD and CD	0 (0)	1 (3.6)	1 (2.0)
Tic disorders	5 (22.7)	7 (25.0)	12 (24.0)
Any disorder	7 (31.8)	17 (60.7)	23 (46.0)

### Sertraline dose, duration, and AEs

Data on the sertraline dose and duration for Step 2 treatment and follow-up are presented in Table 2. The dose was slightly higher for patients who initially received Step 2 CBT before switching to sertraline. At the 12-month follow-up, eight children were still on medication—six from the initial sertraline group and two from the CBT group. By the 24-month follow-up, only four remained on sertraline, and by the 36-month follow-up, all patients had discontinued their medication.

**Table 2:** Mean Sertraline Dose, Duration of Use, and Adverse Events by Treatment Group

	SSRI - Group	CBT+SSRI – Group Step 2 CBT non-responders
Mean dose, posttreatment, M (SD) *N*	128.8 (47.7) 15	142.9 (45.0) 7
FU 12, Mean dose (SD) n	139.3 (55.6) 7	137.5 (53.0) 2
FU 24, Mean dose (SD) n	62.5 (17.7) 2	112.5 (123.7) 2
Months using SRT, M (SD)	14.7 (7.9)	20.0 (12.3)
Participants with moderate AE ≥ 1 during the immediate treatment, % (n)	85 (11)	75 (6)
Participants with moderate AE ≥ 1 during the follow-up period, % (n)	0 (0)	0 (0)
Adverse events, immediate treatment, % (n)		
Gastrointestinal	54 (7)	38 (3)
Psychiatric other than suicidal	62 (8)	38 (3)
Suicidal thoughts or ideation	15 (2)	25 (2)
Activation	15 (2)	25 (2)
Sleep problems	31 (4)	0 (0)
Autonomic	23 (3)	25 (2)
Menstruation	8 (1)	0 (0)
Skin	0 (0)	0 (0)
Tremor	8 (1)	0 (0)
Sexual	8 (1)	0 (0)

Table 2 also reports moderate or severe AEs during both the immediate treatment and follow-up period. No serious AEs were reported. However, 85% of patients in the sertraline group and 75% of patients in the Step 2 CBT + sertraline group experienced at least one moderate or severe AE during the immediate treatment phase. Approximately 15% and 25% of patients, respectively, reported moderate activation symptoms, such as restlessness and impulsivity. In the initial sertraline group, two participants experienced moderate activation effects, which required dose reduction. Nevertheless, both continued treatment. Similarly, in the CBT + sertraline group, one patient experienced activation in the form of impulsivity, leading to a dose reduction. Importantly, no moderate or severe AEs were reported during the follow-up period.

Additionally, a limited number of booster CBT sessions could be offered if necessary during the initial part of the follow-up period. A total of six patients participated in these sessions before the 12-month follow-up. Of these, five patients required one booster session, while one patient received a total of four sessions. These booster sessions were intended to address residual symptoms or reinforce treatment gains during the follow-up phase.

### Continuous outcome

**Fig. 2:**
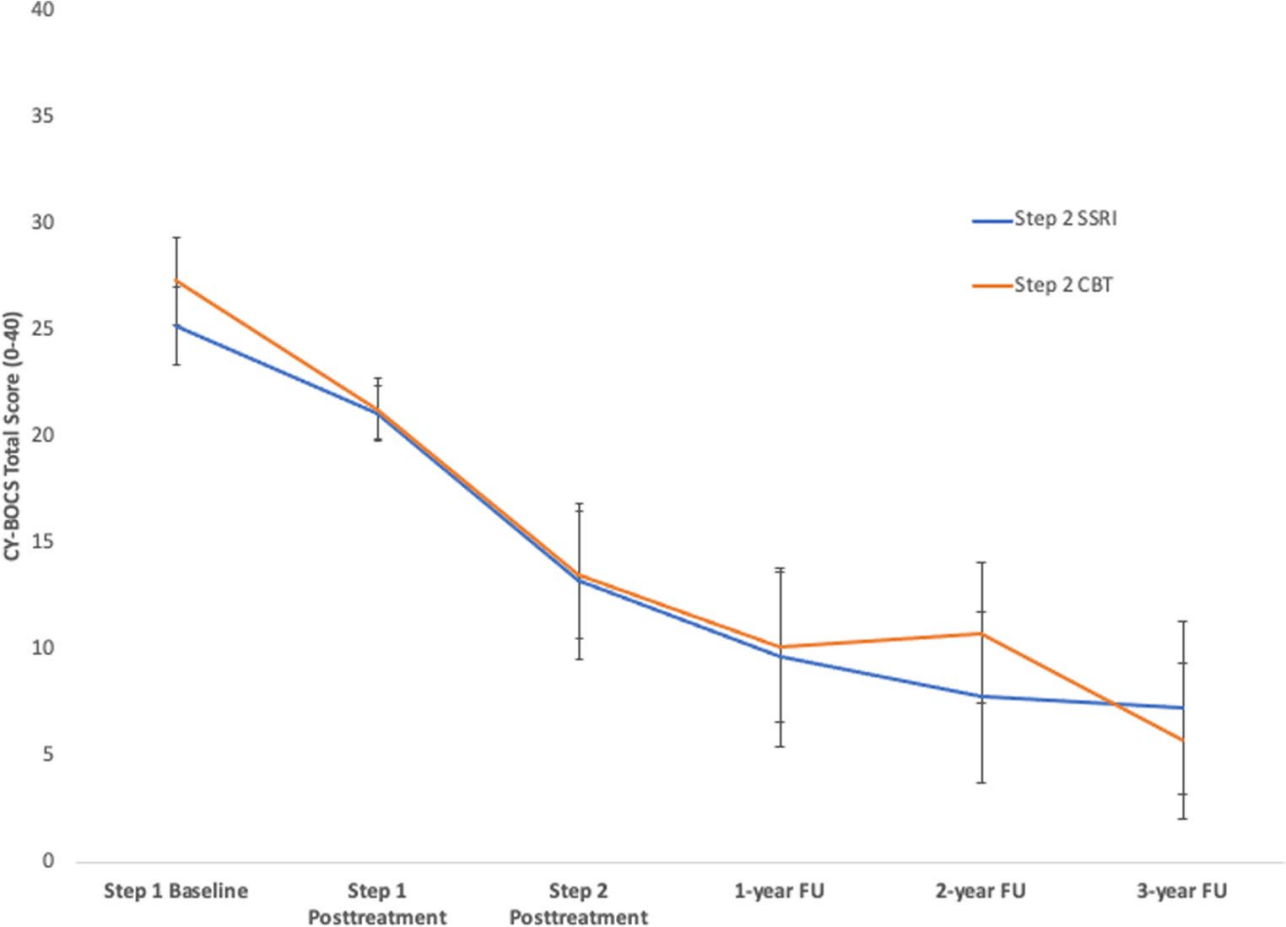
Symptom Trajectories of CY-BOCS Total Scores Across Step 2 Treatment Arms and Follow-Up Assessments

The CY-BOCS total score estimates are presented in Fig. 2; Table 3. Figure 1 illustrates symptom trajectories for both the Step 2 SSRI and Continued CBT from post-treatment through the 3-year follow-up. Both groups experienced a gradual reduction in symptom severity, with no significant differences between the groups at any time point.

**Table 3:** Mild OCD and Clinical Remission Rates, and CY-BOCS Total Score Estimates Across Treatment Groups and Follow-Up

		Continued CBT	*n*/N*	Sertraline	*n*/*N*	Chi-square/t-test
**Mild OCD** **CY-BOCS < 16**						
Step 2 posttreatment		60.9%	14/23	60.0%	9/15	0.00 (0.957)
FU6/after CBT+SSRI		69.6%	16/23	53.3%	8/15	1.03 (0.311)
12 months		80.0%	12/15	81.8%	9/11	0.01 (0.907)
24 months		72.2%	13/18	83.3	10/12	0.50 (0.481)
36 months		93.3%	13/14	91.7%	11/12	0.03 (0.869)
**Clinical remission** **CY-BOCS < 11**						
Step 2 posttreatment		34.8%	8/15	33.3%	5/15	0.00 (0.927)
FU6/after CBT+SSRI		34.8%	8/23	33.3%	5/15	0.00 (0.927)
12 months		66.7%	10/15	54.5%	6/11	0.39 (0.530)
24 months		55.6%	10/18	83.3%	10/12	2.50 (0.114)
36 months		71.4%	10/14	83.3%	10/12	0.39 (0.535)
**CY-BOCS total scores estimates from LME (M**,** SE)**						
Step 2 posttreatment		14.0 (1.31)		12.2 (1.69)		0.85 (0.394)
FU6/after CBT+SSRI		13.4 (1.74)		16.1 (3.99)		−0.61 (0.543)
12 months		9.9 (1.50)		9.3 (1.76)		0.26 (0.796)
24 months		11.0 (1.38)		7.2 (1.69)		1.75 (0.081)
36 months		5.6 (1.50)		6.7 (1.69)		−0.49 (0.625)

* Total number of participants responders/remitters/total number of participants (responders/remitters and non-responders/non-remitters

CBT: 22 finished Step 2 post-treatment assessment. Sertraline: 15 finished Step 2 post-treatment assessment

**Fig. 3:**
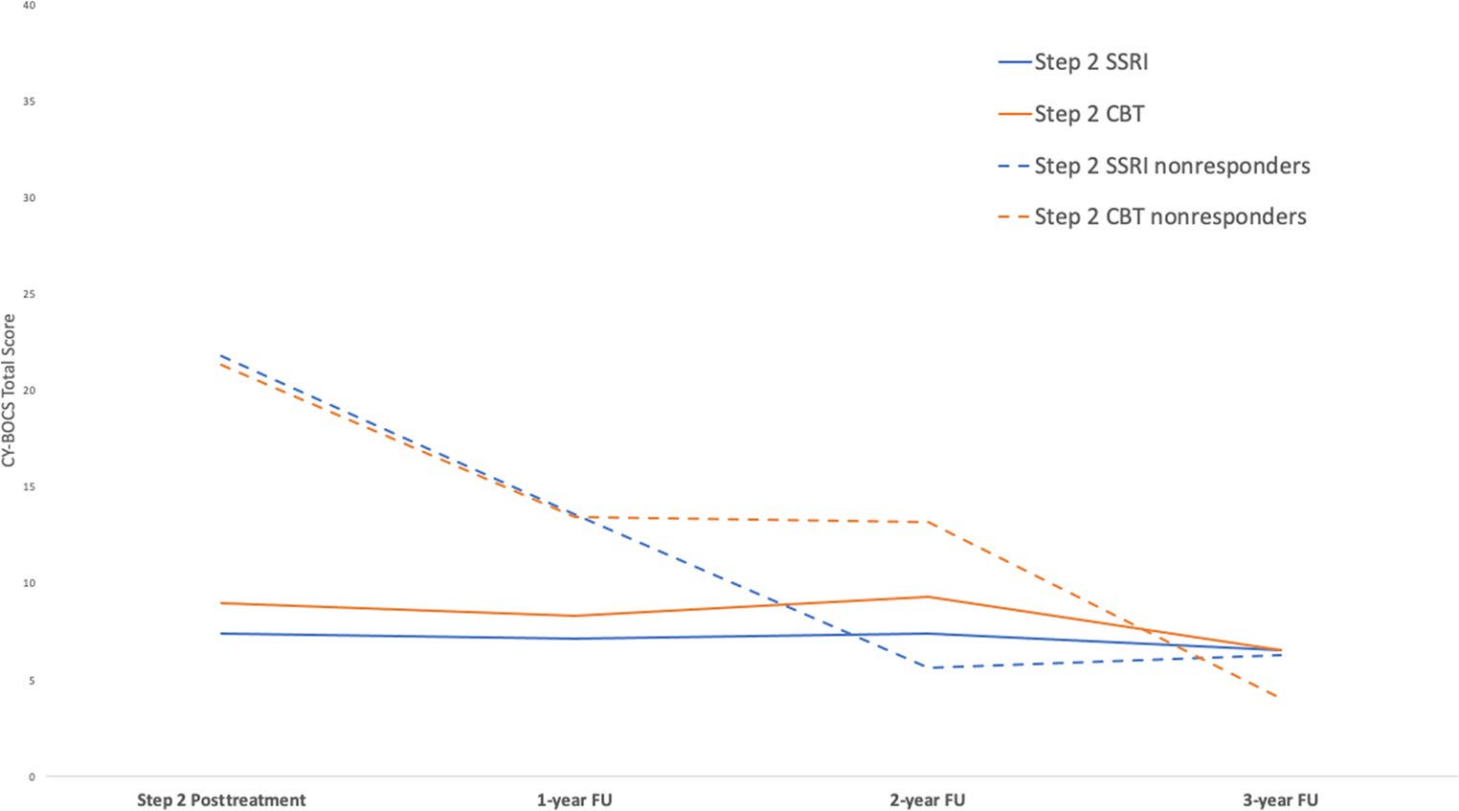
Symptom Trajectories of Responders and Non-Responders Across Step 2 Treatment Arms and Follow-Up Assessments

Figure 3 shows the symptom trajectories for responders and non-responders within both groups. At post-treatment, there is a clear difference in symptom severity between responders and non-responders, particularly for those who received Step 2 CBT. However, this difference diminished over time, and by the 3-year follow-up, there were no significant differences between the groups.

### Categorical outcome

No significant differences between groups were identified regarding categorical outcomes. Overall, at the 3-year follow-up, 92% of the patients had only mild OCD, with 77% achieving remission (see Table 3).

## Discussion

This study examined the long-term outcomes of children and adolescents with OCD who did not respond to initial CBT and were randomized to receive continued CBT or sertraline treatment. It provides the longest follow-up to date of this group, based on the state-of-the-art semi-structured interviews, such as the KSADS-PL for diagnostic assessment and the CY-BOCS for OCD symptom severity. Furthermore, AEs were carefully monitored for all patients treated with sertraline. At the 3-year follow-up, 92% of participants showed considerable improvement: 77% achieved clinical remission and 15% had only mild OCD symptoms, with no significant differences between the continued CBT and sertraline groups. These results underscore the effectiveness of both treatments, indicating that patients can achieve substantial long-term improvements, regardless of the treatment modality used after initial CBT non-response. This highlights the potential of a stepped-care model in treating pediatric OCD, especially those with persistent symptoms following initial treatment.

One interpretation of the findings is that both treatments may facilitate long-term improvement, regardless of the immediate treatment response. Alternatively, the lack of significant group differences might reflect natural symptom fluctuation in this population over time. Without a control group, it remains uncertain whether observed improvements result from the interventions themselves or represent broader developmental trajectories or placebo effects. Another possibility is that medication following a course of CBT operates differently from SSRI treatment alone. While SSRIs primarily alleviate anxiety, CBT provides tools to reframe obsessions as false alarms [42]. It can be suggested that medication was especially beneficial for patients who initially found exposure exercises too challenging, enabling them to better engage with these techniques after anxiety reduction.

Most participants receiving sertraline reported adverse events (AEs), with 85% experiencing at least one moderate or severe AE and 25% indicating moderate activation symptoms, such as restlessness or impulsivity. Importantly, no serious AEs were observed during the study. These results underscore the necessity of close monitoring for pediatric patients receiving SSRIs for OCD, particularly in the context of dose adjustments. Bridge et al. [43] similarly found that SSRI-related activation, including restlessness and impulsivity, was more common in younger children, highlighting the need for careful dose titration and monitoring to mitigate these risks. Despite the relatively high incidence of moderate AEs, most participants tolerated the treatment well enough to continue, suggesting that sertraline remains a feasible option when CBT alone is insufficient.

A standard approach to evaluating whether an AE is treatment-related is by comparing AE rates across all treatment groups. However, comparing AEs becomes more complex if the comparison group receives psychotherapy [44]. Unfortunately, this trial only reported AEs in the group receiving sertraline, making it difficult to determine how these events compare to those experienced in psychotherapy or due to natural symptom fluctuations. Future trials should ensure that AEs are reported across all treatment arms.

Attrition rates during follow-up were 24% at the end of Step 2 and 46% at both the 2- and 3-year follow-ups. While participant attrition is a common challenge in long-term studies, the missing data did not seem to introduce significant bias, as the baseline characteristics of those lost to follow-up were similar to those who completed the assessments. However, the attrition highlights the difficulties of maintaining participant engagement in long-term studies, especially when improvements may result in decreased motivation to stay involved. Innovative strategies to enhance retention and ensure robust long-term data collection are critical for future research.

The improvements may be driven by continued exposure to the principles learned during treatment. The stepped-care model could also enhance outcomes by offering individualized treatment adjustments that address patient-specific barriers to improvement. Further research into the mechanisms underlying the long-term positive outcomes could help refine treatment approaches and identify predictors of sustained success.

Our findings are consistent with previous research supporting the effectiveness of CBT and SSRIs [19, 21, 22]. In contrast to earlier studies indicating poorer prognoses [17], our results show that significant improvements can be achieved and sustained over time with a comprehensive treatment approach. The high remission rate in our study further supports the benefits of combining CBT with sertraline when necessary, especially for those who do not respond to initial treatment. However, recent advancements in treatment, such as online CBT programs [45–47] or more concentrated applications of CBT [48, 49], or sensor-assisted CBT [50], may provide additional benefits. The latter integrates real-time monitoring of physiological responses to exposures, allowing for better adjustment of difficulty and engagement, which may enhance treatment effectiveness. Studies comparing traditional stepped-care models with these emerging modalities could inform future guidelines.

To build on these findings, future studies could explore predictors and moderators of treatment response and remission, such as patient characteristics or biological markers, to identify subgroups most likely to benefit from specific interventions. Randomized controlled trials with placebo or alternative control groups could also clarify the specific effects of treatment compared to natural symptom fluctuations. Additionally, long-term studies evaluating newer therapeutic approaches, including adjunctive digital tools or personalized pharmacotherapy, are needed to expand treatment options for non-responders.

Future studies should aim to replicate this study in larger samples to strengthen statistical power and the feasibility of conducting predictor or moderator analyses of treatment outcome. Evaluating the cost-effectiveness of stepped-care approaches would also be valuable. Furthermore, research in more diverse clinical and cultural contexts is needed to determine the generalizability and scalability of the current findings across different systems of healthcare. 

### Strengths and limitations

This study has several strengths, especially the robust longitudinal design, which allowed us to track symptom trajectories over three years, and the use of validated semi-structured tools such as the CY-BOCS and K-SADS-PL. Additionally, our sample was large and spanning three Nordic countries. The stepped-care model used in this trial offers valuable insight into the treatment pathways for children with OCD, especially for those who do not respond to initial CBT. However, several limitations should be noted. First, the relatively high rate of attrition may limit the generalizability of our findings. Second, the sample size in Step 2 (*N* = 50) was relatively small, particularly in terms of subgroup analysis. This modest sample size also limited the statistical power to detect small or moderate between-group effects. Consequently, the absence of significant group differences should be interpreted with caution, as the study may have been underpowered to identify such effects. Nevertheless, the consistent with-in group improvements and stable long-term outcomes across both treatments provide evidence of clinical benefit despite these constraints. Missing data could also have affected the accuracy of our longitudinal findings, despite using statistical methods to address this issue. Lastly, the absence of a control group in Step 2 prevents definitive conclusions about the treatment effects compared to natural symptom fluctuation or placebo response.

Although exploratory moderator or predictor analyses (e.g., by age, baseline severity, or comorbidity) would be clinically informative, the present sample size of Step 2 limits the feasibility of such analyses. Future studies with larger samples should explore potential predictors or moderators of long-term outcome to improve personalized treatment planning.

## Conclusion

This study highlights the long-term effectiveness of a stepped-care model for children and adolescents with OCD who do not respond to initial CBT. At the 3-year follow-up, 92% of participants exhibited only mild OCD symptoms, and 77% achieved remission. The lack of group differences suggests that treatment decisions should be individualized, considering patient preference and clinical needs. Given the high rate of symptom improvement over time, future studies should further explore mechanisms driving long-term outcomes and strategies to enhance treatment delivery, ensuring it remains both effective and efficient.

## Supplementary Information

Below is the link to the electronic supplementary material.

Supplementary Material 1 (DOCX 34.4 KB)

## Data Availability

The clinical data used in this study were collected in the Nordic Longterm OCD Treatment Study. Due to privacy regulations and ethical restrictions, these data are not publicly available and cannot be shared.
