# Supplementary material for: Three-Year Follow-Up of children and adolescents with OCD Who Did Not Respond to Initial Cognitive-Behavioral Therapy (CBT): Outcomes of Continued CBT vs. Sertraline
Source: Eur Child Adolesc Psychiatry. 2026 Mar 19;35(7):2155–66. doi: 10.1007/s00787-026-03009-3 (PMC13427785; doi:10.1007/s00787-026-03009-3)
Supplement: Supplementary file 1 — Supplementary Material 1 (DOCX 34.4 KB) [file 787_2026_3009_MOESM1_ESM.docx]

Step 2 FU supplemental tables

Table S1

Patient Characteristics, Comorbid Diagnoses, and Main OCD Symptoms by Treatment Response Status

|  | Total | SSRI responders | SSRI nonresponders | CBT responders | CBT nonresponders |
| --- | --- | --- | --- | --- | --- |
| Gender, % (n) |  |  |  |  |  |
| Female | 52 (26) | 33 (3) | 67 (4) | 57 (8) | 56 (5) |
| Male | 48 (24) | 67 (6) | 33 (2) | 43 (6) | 44 (4) |
| Age, M (SD) | 13.1 (2.8) | 12.1 (2.8) | 13.0 (3.2) | 12.6 (3.7) | 13.2 (2.9) |
| Age of onset, M (SD) | 7.8 (3.5) | 9.1 (2.6) | 5.2 (2.9) | 7.6 (4.2) | 7.7 (3.0) |
| CYBOCS baseline, M (SD) |  |  |  |  |  |
| Obsession | 13.0 (3.0) | 12.7 (2.5) | 11.7 (2.5) | 13.4 (2.9) | 13.3 (2.6) |
| Compulsion | 13.3 (2.9) | 12.4 (2.7) | 13.3 (2.3) | 13.1 (3.3) | 13.7 (2.3) |
| Total score | 26.3 (5.6) | 25.1 (4.9) | 25.0 (4.6) | 26.4 (6.1) | 27.0 (4.6) |
| Comorbidity, % (n) |  |  |  |  |  |
| Depressive disorders | 6 (3) | 0 (0) | 0 (0) | 14 (2) | 11 (1) |
| Anxiety disorders | 4 (12) | 11 (1) | 33 (2) | 14 (2) | 56 (5) |
| ADHD | 14 (7) | 11 (1) | 17 (1) | 21 (3) | 11 (1) |
| Disruptive disorders | 2 (1) | 0 (0) | 0 (0) | 7 (1) | 0 (0) |
| Tic disorders | 20 (10) | 22 (2) | 17 (0) | 14 (2) | 44 (4) |
| OCD symptoms, % (n) |  |  |  |  |  |
| Checking Compulsions | 40 (20) | 33 (3) | 17 (1) | 71 (10) | 67 (6) |
| Hoarding Obsessions/Compulsions | 18 (9) | 33 (3) | 17 (1) | 14 (2) | 33(3) |
| Contamination Obsessions/Cleaning Compulsions | 50 (25) | 67 (6) | 50 (3) | 79 (11) | 56 (5) |
| Aggressive Obsessions | 30 (15) | 44 (4) | 33 (2) | 29 (4) | 56 (5) |
| Religious Obsessions | 6 (3) | 22 (2) | 17 (1) | 0 (0) | 0 (0) |
| Symmetry Obsessions/Compulsions | 22 (11) | 11 (1) | 50 (3) | 29 (4) | 33 (3) |
| Repeating Compulsions | 18 (9) | 22 (2) | 17 (1) | 7 (1) | 56 (5) |
| Sexual Obsessions | 12 (6) | 11 (1) | 33 (2) | 7 (1) | 22 (2) |
| Magical Obsessions/Compulsions | 2 (1) | 11 (1) | 0 (0) | 0 (0) | 0 (0) |
| Somatic Obsessions | 4 (2) | 0 (0) | 0 (0) | 7 (1) | 11 (1) |
| Counting Compulsions | 2 (1) | 0 (0) | 0 (0) | 7 (1) | 0 (0) |

Table S2

Individual Symptom Severity Trajectories and Percent Reductions in CY-BOCS Total Scores Across Treatment Steps and Follow-Up

|  | Case no. | Step 1 baseline | Step 1 post/2 baseline | % reduction step 1 | Step 2, post | % Reduction step 2 | % Reduction step 1-Step 2 | FU 6 / Step 2 CBT+SSRI | FU 12 | FU 24 | FU 36 |
| --- | --- | --- | --- | --- | --- | --- | --- | --- | --- | --- | --- |
| SSRI responders | 1 | 20 | 18 | 10,0 | 6 | 66,7 | 70,0 |  | 12 | 9 | 9 |
|  | 2 | 26 | 25 | 3,8 | 0 | 100,0 | 100,0 |  | 0 | 9 | 8 |
|  | 3 | 23 | 19 | 17,4 | 11 | 42,1 | 52,2 | 19 | 11 | 7 | 9 |
|  | 4 | 21 | 21 | 0,0 | 12 | 42,9 | 42,9 |  | 8 | 10 | 3 |
|  | 5 | 28 | 19 | 32,1 | 4 | 78,9 | 85,7 |  | 0 | 0 | 0 |
|  | 6 | 27 | 17 | 37,0 | 11 | 35,3 | 59,3 |  | 16 | 16 | 16 |
|  | 7 | 23 | 16 | 30,4 | 11 | 31,3 | 52,2 | 11 | 8 | 8 | 0 |
|  | 8 | 22 | 23 | -4,5 | 10 | 56,5 | 54,5 |  | 3 | 0 | 6 |
|  | 9 | 36 | 27 | 25,0 | 2 | 92,6 | 94,4 |  |  | 8 | 8 |
| SSRI nonresponders | 10 | 27 | 20 | 25,9 | 21 | -5,0 | 22,2 |  | 21 | 16 | 11 |
|  | 11 | 25 | 22 | 12,0 | 22 | 0,0 | 12,0 |  | 5 | 0 | 7 |
|  | 12 | 26 | 20 | 23,1 | 20 | 0,0 | 23,1 |  | 14 | 0 | 0 |
|  | 13 | 21 | 18 | 14,3 | 19 | -5,6 | 9,5 |  |  |  |  |
|  | 14 | 19 | 24 | -26,3 | 22 | 8,3 | -15,8 |  |  |  |  |
|  | 15 | 32 | 20 | 37,5 | 27 | -35,0 | 15,6 |  |  |  |  |
| CBT responders | 16 | 20 | 16 | 20,0 | 8 | 50,0 | 60,0 |  | 8 | 24 |  |
|  | 17 | 34 | 23 | 32,4 | 0 | 100,0 | 100,0 | 0 |  |  |  |
|  | 18 | 24 | 17 | 29,2 | 12 | 29,4 | 50,0 |  | 19 | 10 |  |
|  | 19 | 31 | 17 | 45,2 | 9 | 47,1 | 71,0 |  | 1 | 0 | 0 |
|  | 20 | 26 | 18 | 30,8 | 14 | 22,2 | 46,2 |  | 19 | 19 | 15 |
|  | 21 | 30 | 22 | 26,7 | 0 | 100,0 | 100,0 |  | 8 | 0 | 0 |
|  | 22 | 18 | 21 | -16,7 | 10 | 52,4 | 44,4 |  |  | 8 | 7 |
|  | 23 | 23 | 17 | 26,1 | 9 | 47,1 | 60,9 |  |  | 7 | 17 |
|  | 24 | 25 | 23 | 8,0 | 9 | 60,9 | 64,0 | 9 | 6 | 23 |  |
|  | 25 | 22 | 18 | 18,2 | 15 | 16,7 | 31,8 |  | 9 | 2 | 0 |
|  | 26 | 34 | 19 | 44,1 | 14 | 26,3 | 58,8 |  | 8 | 18 | 14 |
|  | 27 | 32 | 22 | 31,3 | 12 | 45,5 | 62,5 |  | 10 |  |  |
|  | 28 | 34 | 31 | 8,8 | 14 | 54,8 | 58,8 |  | 5 | 3 | 0 |
|  | 29 | 17 | 18 | -5,9 | 0 | 100,0 | 100,0 | 0 | 0 | 0 | 0 |
| CBT nonresponders | 30 | 29 | 22 | 24,1 | 17 | 22,7 | 41,4 | 14 | 14 | 10 | 0 |
|  | 31 | 23 | 20 | 13,0 | 25 | -25,0 | -8,7 | 20 |  | 14 | 9 |
|  | 32 | 21 | 20 | 4,8 | 21 | -5,0 | 0,0 | 11 |  | 11 | 4 |
|  | 33 | 31 | 21 | 32,3 | 22 | -4,8 | 29,0 | 20 | 1 | 19 | 0 |
|  | 34 | 28 | 20 | 28,6 | 16 | 20,0 | 42,9 | 18 | 14 | 10 | 12 |
|  | 35 | 36 | 27 | 25,0 | 24 | 11,1 | 33,3 | 20 | 23 | 14 | 2 |
|  | 36 | 24 | 23 | 4,2 | 24 | -4,3 | 0,0 | 21 |  |  |  |
|  | 37 | 24 | 17 | 29,2 | 21 | -23,5 | 12,5 | 16 |  |  |  |
|  | 38 | 27 | 28 | -3,7 | 25 | 10,7 | 7,4 |  |  |  |  |

Table S3

SRT dose, duration, and adverse events (AE).

|  | Case no. | Final dose of SRT at posttreatment | Dose at FU12 | Dose at FU24 | Months on SRT | AE Step 2 (mod/sev) | AE FU mod/sev | Other meds stable dose |
| --- | --- | --- | --- | --- | --- | --- | --- | --- |
| SSRI responders | 1 | 75 | - | - | 7 | Psychiatric and sleep |  |  |
|  | 2 | 100 | - | - | 7 | Psychiatric |  |  |
|  | 3 | N/A | n/a |  | N/A |  |  |  |
|  | 4 | 75 | - | - | 7 | Gastrointestinal, psychiatric, suicidal ideation, activation, sleep |  |  |
|  | 5 | 100 | 50 | - | 12 | Gastrointestinal, psychiatric, suicidal ideation, activation, autonomic | None |  |
|  | 6 | 150 | 150 | 50 | 25 | Autonomic, sexual | None |  |
|  | 7 | 175 | 175 | - | 22 | Gastrointestina |  |  |
|  | 8 | 75 | 75 | 75 | 28 | None |  | Concerta 54 mg (only at FU) |
|  | 9 | 75 | - | - | 7 | Gastrointestinal, autonomic, tremor |  |  |
| SSRI nonresponders | 10 | 150 | 175 | - | 21 | Gastrointestinal, psychiatric, menstrual | None |  |
|  | 11 | 200 | 150 | - | 13 | Gastrointestinal, psychiatric | None | Concerta 27 mg x 1, Aripiprazole 5 mg (only at FU) |
|  | 12 | 150 | 200 | - | 13 | Gastrointestinal, psychiatric |  |  |
|  | 13 | N/A | n/a |  | N/A | Psychiatric, sleep |  |  |
|  | 14 | 200 | n/a |  | N/A | none |  |  |
|  | 15 | 150 | n/a |  | N/A |  |  |  |
| CBT responders | 16 | 125 | - | - | 6 |  |  |  |
|  | 17 |  |  |  |  |  |  |  |
|  | 18 |  |  |  |  |  |  |  |
|  | 19 |  |  |  |  |  |  |  |
|  | 20 |  |  |  |  |  |  |  |
|  | 21 |  |  |  |  |  |  |  |
|  | 22 |  |  |  |  |  |  |  |
|  | 23 |  |  |  |  |  |  |  |
|  | 24 |  |  |  |  |  |  |  |
|  | 25 |  |  |  |  |  |  |  |
|  | 26 |  |  |  |  |  |  |  |
|  | 27 |  |  |  |  |  |  |  |
|  | 28 |  |  |  |  |  |  |  |
|  | 29 |  |  |  |  |  |  |  |
| CBT nonresponders | 30 |  | n/a | n/a | N/A | Psychiatric, suicidal ideation, activation, autonomic |  |  |
|  | 31 | 200 | n/a | n/a | N/A | psychiatric |  |  |
|  | 32 | 200 | n/a | n/a | N/A | Gastrointestinal |  |  |
|  | 33 | 150 | 175 | 25 | 21 | Gastrointestinal | None |  |
|  | 34 | 150 | - | - | 6 | None |  | Melatonin 3mg till natten |
|  | 35 | 100 | 100 | 200 | 29 | None |  |  |
|  | 36 | 100 (4th visit last visit) | n/a | n/a | N/A | Psychiatric, suicidal ideation, activation |  | Strattera 18 x 3. Melatonin 3 mg x 2. |
|  | 37 | 100 | n/a | n/a | N/A | Gastrointestinal |  |  |
|  | 38 |  | n/a | n/a | N/A |  |  |  |

Table S4

Estimated CY-BOCS total score across Follow-up Assessment for CBT and SRT participants

|  |  |  | 95% CI |  |
| --- | --- | --- | --- | --- |
|  | Mean | SE | Lower Bound | Upper Bound |
| CBT |  |  |  |  |
| Baseline | 27,4 | 1,1 | 25,1 | 29,6 |
| After Step 1 CBT | 21,3 | 1,1 | 19,1 | 23,5 |
| After Step 2 | 14,0 | 1,3 | 11,4 | 16,6 |
| 1-y FU | 9,9 | 1,5 | 6,9 | 12,8 |
| 2-y FU | 11,0 | 1,4 | 8,3 | 13,8 |
| 3-y FU | 5,6 | 1,5 | 2,6 | 8,6 |
| SRT |  |  |  |  |
| Baseline | 25,2 | 1,3 | 22,7 | 27,7 |
| After Step 1 CBT | 21,4 | 1,3 | 18,9 | 23,9 |
| After Step 2 | 12,2 | 1,7 | 8,9 | 15,5 |
| 1-y FU | 9,3 | 1,8 | 5,8 | 12,8 |
| 2-y FU | 7,2 | 1,7 | 3,9 | 10,5 |
| 3-y FU | 6,7 | 1,7 | 3,4 | 10,0 |

Table S5

Estimated CY-BOCS total score across Follow-up Assessment for CBT and SRT responders and nonresponders

|  |  |  | 95% CI |  |
| --- | --- | --- | --- | --- |
|  | Mean | SE | Lower Bound | Upper Bound |
|  |  |  |  |  |
| CBT responders |  |  |  |  |
| Baseline | 26,4 | 1,4 | 23,6 | 29,2 |
| After Step 1 CBT | 20,1 | 1,4 | 17,4 | 22,9 |
| Step 2 | 9,5 | 1,5 | 6,6 | 12,4 |
| 1-y FU | 8,3 | 1,6 | 5,2 | 11,4 |
| 2-y FU | 9,6 | 1,5 | 6,6 | 12,6 |
| 3-y FU | 6,1 | 1,7 | 2,7 | 9,6 |
| CBT non-responders |  |  |  |  |
| Baseline | 27,8 | 1,9 | 24,1 | 31,4 |
| After Step 1 CBT | 21,9 | 1,9 | 18,2 | 25,6 |
| Step 2 | 21,9 | 2,0 | 18,0 | 25,8 |
| 1-y FU | 12,7 | 2,6 | 7,5 | 17,8 |
| 2-y FU | 13,0 | 2,1 | 8,8 | 17,2 |
| 3-y FU | 4,5 | 2,1 | 0,3 | 8,7 |
| SRT responders |  |  |  |  |
| Baseline | 25,1 | 1,8 | 21,6 | 28,6 |
| After Step 1 CBT | 20,6 | 1,8 | 17,1 | 24,0 |
| Step 2 | 7,0 | 1,9 | 3,3 | 10,7 |
| 1-y FU | 7,4 | 1,9 | 3,7 | 11,1 |
| 2-y FU | 7,4 | 1,8 | 4,0 | 10,9 |
| 3-y FU | 6,6 | 1,8 | 3,1 | 10,0 |
| SRT non-responders |  |  |  |  |
| Baseline | 25,0 | 2,2 | 20,7 | 29,3 |
| After Step 1 CBT | 20,7 | 2,2 | 16,4 | 24,9 |
| Step 2 | 22,3 | 2,6 | 17,1 | 27,5 |
| 1-y FU | 13,4 | 3,0 | 7,4 | 19,3 |
| 2-y FU | 5,4 | 3,0 | -0,6 | 11,3 |
| 3-y FU | 6,0 | 3,0 | 0,1 | 12,0 |
